# Osteogenesis imperfecta and clubfoot—a rare combination: Case report and review of the literature

**DOI:** 10.1097/MD.0000000000004505

**Published:** 2016-08-07

**Authors:** Pietro Persiani, Filippo Maria Ranaldi, Lorena Martini, Anna Zambrano, Mauro Celli, Patrizia D’Eufemia, Ciro Villani

**Affiliations:** a Universitary Department of Anatomic, Histologic, Forensic and Locomotor Apparatus Sciences—Section of Locomotor Apparatus Sciences, Sapienza University of Rome; b Center for Congenital Osteodystrophies, Pediatric Department—Policlinico Umberto I, Italy.

**Keywords:** Bisphosphonates, Clubfoot, Congenital talipes equinovarus, Deformities, Osteogenesis imperfecta, Posteromedial release

## Abstract

**Background:**

Osteogenesis imperfecta (OI) is a rare congenital genetic osteodystrophy, which has a prevalence of 1:20,000. OI is caused by the mutation of the COL1A1/COL1A2 genes, leading to a deficit of quality and/or quantity in the synthesis of procollagen-α type 1. Seven different forms of diverse clinical entity have been classified by Sillence and Glorieux, although, recently, up to 11 forms characterized by different genetic mutations have been recognized. Patients with OI suffer from extreme bone fragility and osteoporosis, which often predisposes them to frequent fractures. This paper presents the case of a child with OI type IV who, at birth, was also diagnosed with a severe clubfoot (congenital talipes equinovarus) grade III. Patient's mother also suffers from OI type IV.

**Methods:**

The treatment was started by placing femoro-podalic corrective casts, according to the Ponseti method, but some unexpected problems occurred during this treatment. When the patient was 3 months of age, we decided to correct the clubfoot before the time limit planned, performing a bilateral posteromedial surgical release.

**Results:**

Three weeks after surgery the casts were removed and replaced with bilateral Spica cast-like braces. On the 6th postoperative week, the patient began wearing Bebax corrective shoes, after 1 year ambidextrous orthopedic shoes. Now, he is 2 years old and has started to walk properly without any orthesis.

**Conclusion:**

In the presence of an orthopedic pathology associated with OI, it is recommended to manage the patient according to the underlying pathology, always considering the bone fragility associated with OI. The final surgical treatment to correct the clubfoot can be done earlier, if necessary. In our opinion, this uncommon association between OI and clubfoot is non-syndromic. This means that the two congenital diseases are not necessarily included in a singular uncommon genetic syndrome, but the clubfoot was caused by multifactorial causes, especially by both the mother's bisphosphonate drug therapy and the amniocentesis performed during her pregnancy to drain polyhydramnios. In our analysis, those environmental factors could have interacted with an already altered genetic substratum, contributing to develop this rare combination of congenital disorders.

## 1 Introduction

Osteogenesis imperfecta (OI) is a rare congenital genetic osteodystrophy. This mutation leads to a quality and/or quantity deficit in the bones of patients with OI, characterized by extreme bone fragility and osteoporosis, which often predisposes them to frequent fractures.

In addition, according to the classification of Sillence and Glorieux (2004), 7 different OI forms of diverse clinical entity have been classified, although, recently, up to 11 forms characterized by different genetic mutations have been recognized.^[1–3]^ The prevalence of OI cases in the world's population is approximately 1:20,000 livebirths,^[4]^ of which about half are affected with OI type I or IV.

**Figure 1:**
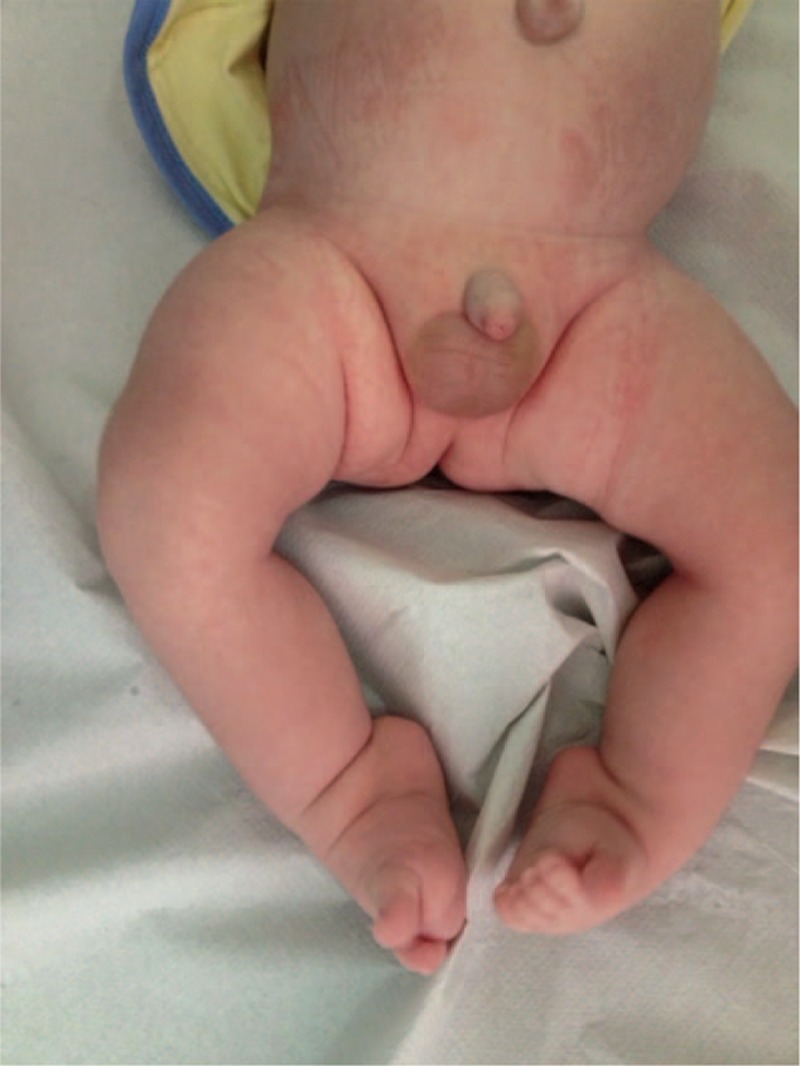
Uncommon III grade clubfoot (congenital talipes equinovarus [CTE]) in newborn patient affected by OI. OI = osteogenesis imperfecta.

The authors report the case of a newborn suffering from OI type IV, who was brought to us for observation, at the Center for Reference and Treatment of Congenital Osteodystrophies, located in the Department of Pediatrics of our hospital, at the age of 2 weeks. The patient had a clubfoot (congenital talipes equinovarus [CTE]) grade III (Fig. 1), in combination with the basic genetic disorder. The clubfoot deformity has a prevalence of 1:1000 livebirths,^[5]^ and this epidemiological data can give a clear picture of how rare it is to find a combination of these two pathologies. On the other hand, OI type IV is an autosomal dominant condition, compatible with life even if its quality could be poor. This disease is caused by the mutation of the COL1A1/COL1A2 genes, leading to a deficit of quality and/or quantity in the synthesis of the procollagen-α type 1.

This is an OI type of moderate seriousness and patients affected are characterized by low height, mild or moderate scoliosis, important bone weakness, white/grey sclerae, and dentinogenesis imperfecta.

Our patient's mother, who also suffers from OI type IV, had given birth to her child at the 34th week of gestation and reported a history of other two pregnancies brought to term, three bouts of polyhydramnios during the pregnancy, and the voluntary suspension of the infusion therapy with neridronate for the last 2 years. All her children were affected with OI, but no other had presented with clubfoot.

## 2 Materials and methods

According to Italian law, it is not necessary that an ethical committee approve this kind of study even if the patient is a minor, because an informed consent describing the study purpose was approved and signed by the patient's parents, before starting the study.

The treatment was started after 2 weeks from the patient's birth, by placing lightweight femoro-podalic corrective casts, according to the Ponseti method, to be changed every 2 weeks in order to then proceed to the surgical correction of any residual deformities. As the patient's parents had to commute from another region, which was rather far from our Center for Reference and Treatment of Congenital Osteodystrophies in the Pediatrics Department of the Policlinico Umberto I Hospital, there was no possibility of a strict control on the progress of this treatment.

**Figure 2:**
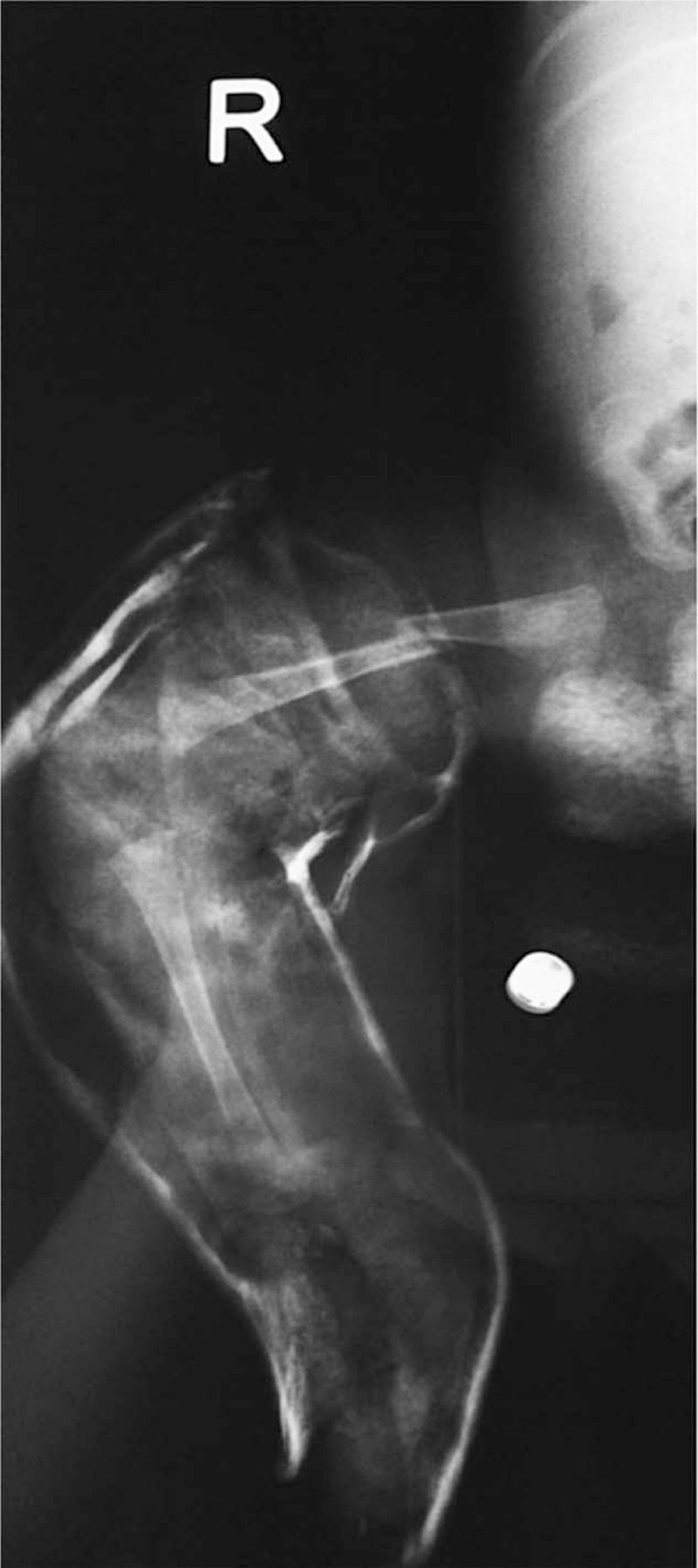
Right femur shaft fracture, accidentally caused by a leverage effect of the cast on the femur of the child.

After 2 weeks of treatment with the casts, when the baby was 1 month of age, we were faced with the first unexpected problem: a shaft fracture at the middle third of the right femur, caused by a leverage effect of the cast on the femur of the child, which happened when his mother picked him up without supporting the leg (Fig. 2). A closed reduction of the fracture under anaesthesia was performed, with pelvic-podalic Spica casting, including the feet, maintaining the correction of the clubfoot.

**Figure 3:**
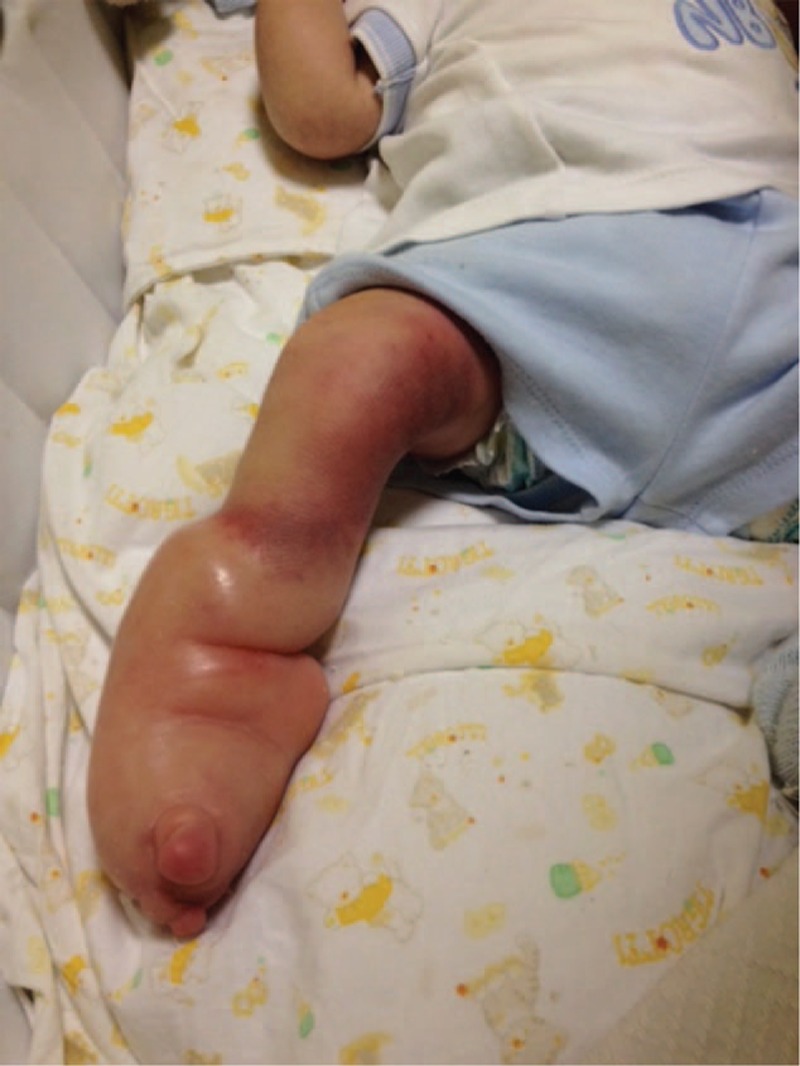
Venturi vacuum effect on the baby's foot, caused by the parent's cutting of the cast just above the foot.

About 21 days after the reduction, a second problem presented itself. Without medical consultation, the parents decided to cut the cast just above the foot, as they judged it to be too tight and that it could cause pain to the child. This decision resulted in a Venturi vacuum effect on the baby's foot, which swelled with a massive deposit of liquids (Fig. 3). The Venturi vacuum effect is the phenomenon that occurs when a fluid that flows through a circular duct is forced through a narrow section, resulting in a pressure reduction and a velocity increase. This effect determines that, in the constricted circular duct, the fluid's hydrostatic pressure decreases and its velocity increases on the distal side of the constriction.^[6]^

Faced with such third party handling of the child's care, both due to the distance and the difficulty by part of parents to handle such a delicate situation, and as the diaphyseal fracture of the femur was in advanced state of consolidation, we decided to wait at least 15 days, allowing the swelling of the foot to go down, in order to then intervene surgically, ahead of the predicted schedule, to correct the clubfoot.

The femoral shaft fracture healed in 45 days from the injury, without any kind of remaining deformity; the bone callus formation was normal, without any delayed union or vicious consolidation.

Consequently, when the patient was 3 months old, we performed a bilateral posteromedial release modified technique. This is a syndesmoplasty and tenoplasty surgical procedure, which consisted in: the lengthening of the Achilles tendon, the posterior tibial tendons, the flexor digitorum communis, and flexor hallucis; the opening of the subtalar, talonavicular, and medial cuneiform joints in order to correct any deformities.

## 3 Results

**Figure 4:**
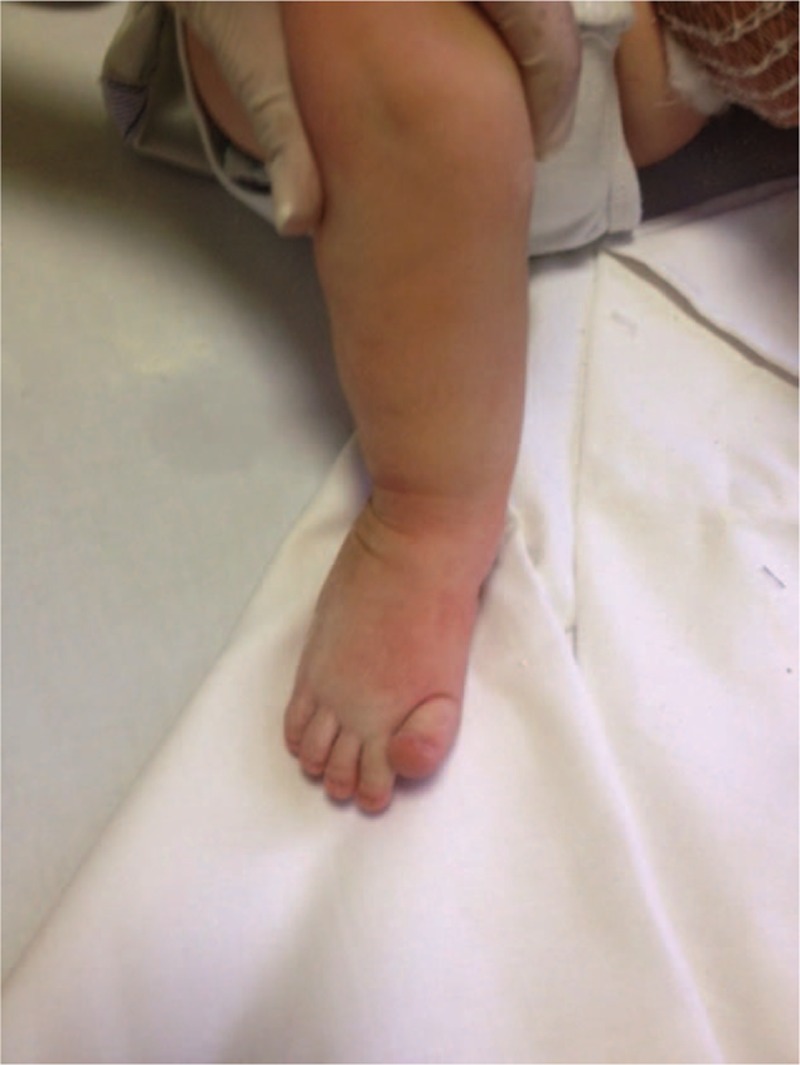
Cast removal and right foot correction 3 weeks after surgery.

**Figure 5:**
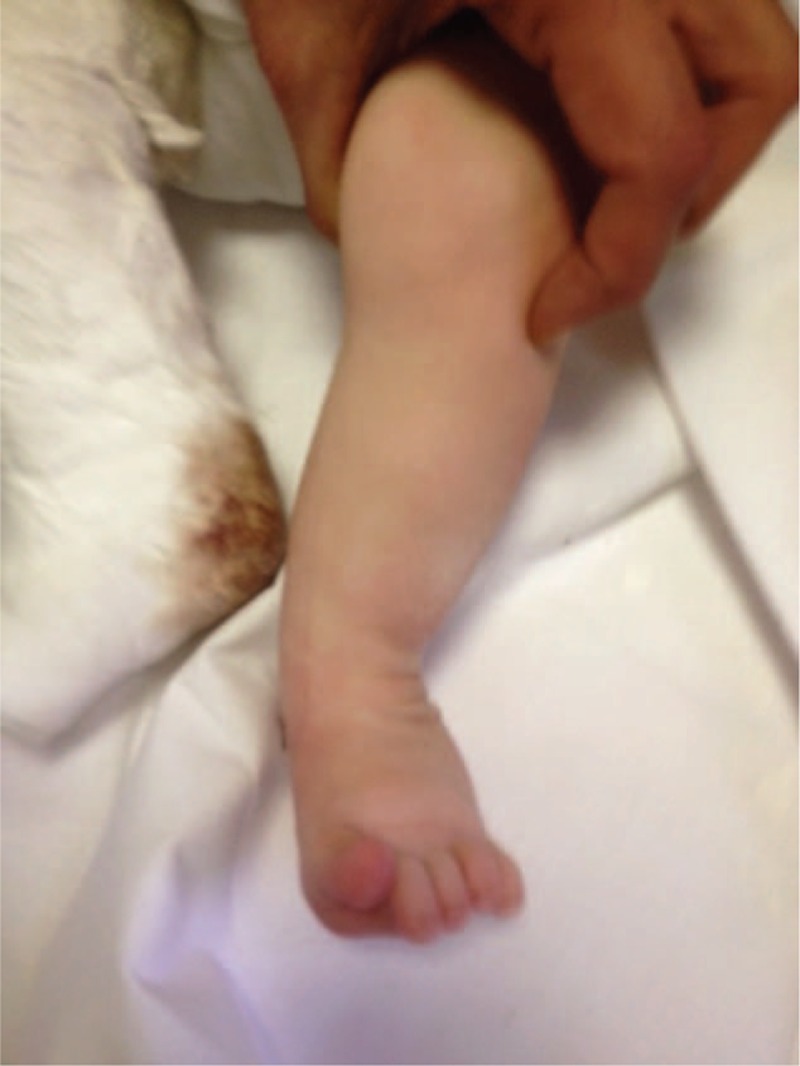
Cast removal and left foot correction 3 weeks after surgery.

**Figure 6:**
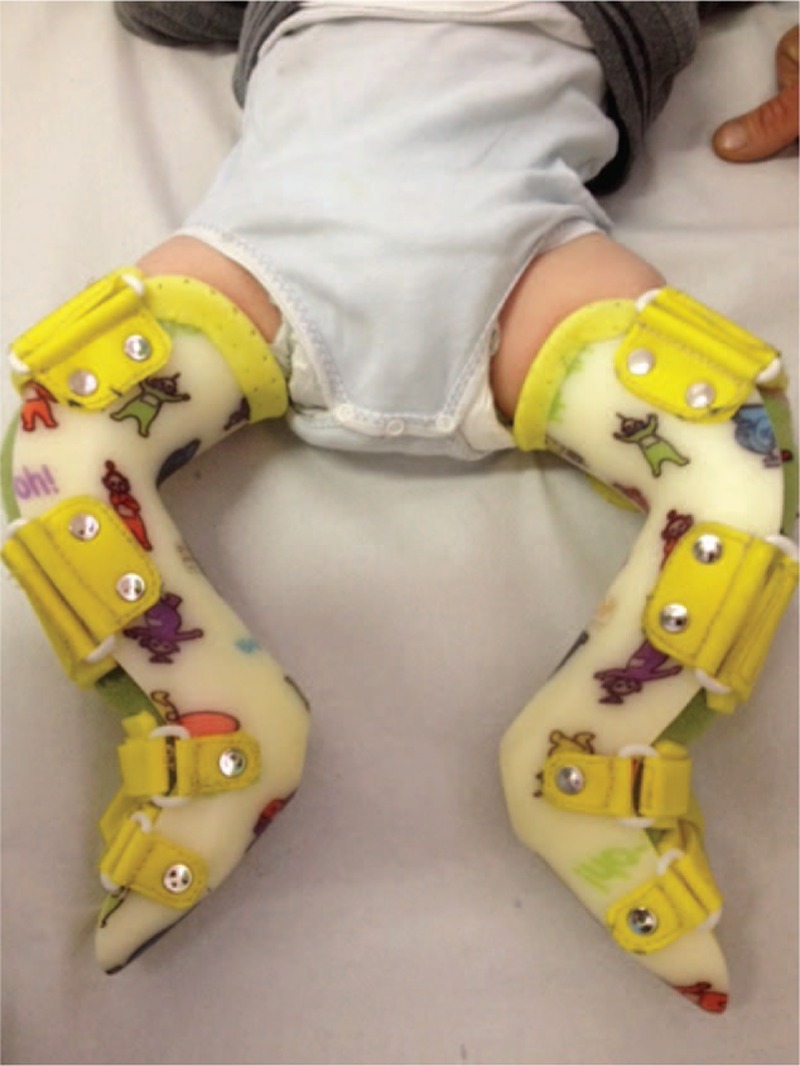
Bilateral spica cast-like braces application to maintain the corrections obtained with surgery.

Three weeks after surgery the casts were removed and replaced with bilateral Spica cast-like braces, in order to maintain the obtained correction (Figs. 4–6). On the 6th postoperative week, the patient, now almost 5 months old, began wearing Bebax corrective shoes (CAMP^®^) 24 hours per day for 9 months.^[7]^ Then, in the next 4 months, Bebax corrective shoes were worn only during the night, interchanging them with ambidextrous orthopedic shoes during the day. The patient, at the age of 18 months, started to wear only orthopedic shoes. There were no further complications.

**Figure 7:**
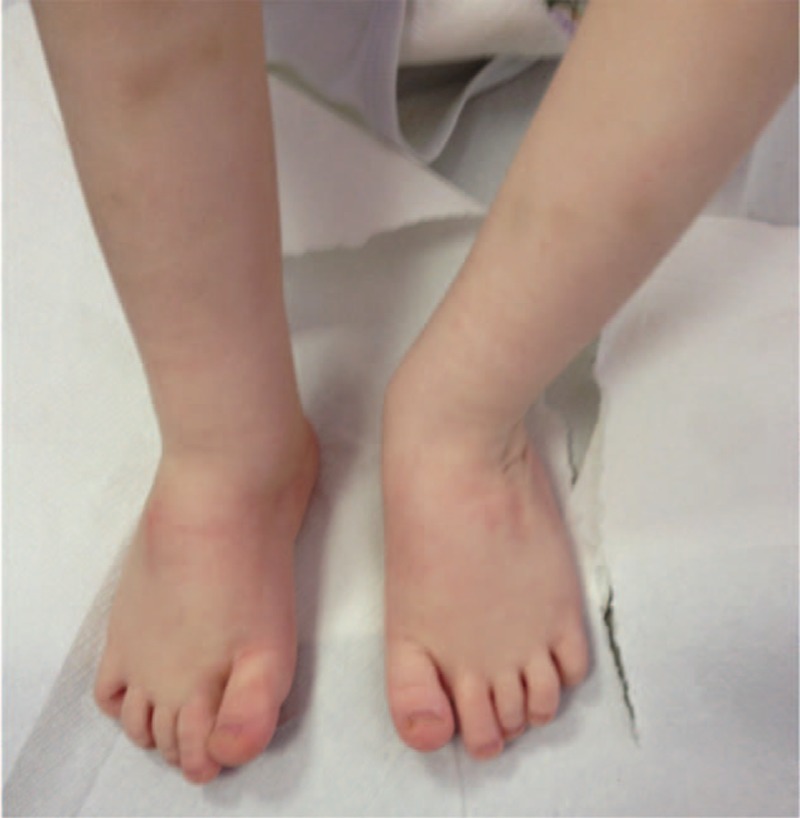
Clinical control 2 years after surgery: the patient walks properly, without pain, and with a minimum residual adduction of the forefoot.

The patient has now turned 2 years of age, and ambulates properly without pain and with a minimum residual adduction of the forefoot, but without any stiffness (Fig. 7). He is continuing a medical therapy with neridronate (therapeutic dose: 2 mg/kg i.v. every 3 months) at our center, according to the OI protocol of treatment. The patient is followed and controlled periodically in our Center for Reference and Treatment of Congenital Osteodystrophies, in the Pediatrics Department of the Policlinico Umberto I Hospital.

## 4 Discussion

In medical literature, we searched for how many cases of clubfoot (CTE) combined with OI were reported. The research was done on MEDLINE using the following keywords.

“Osteogenesis imperfecta,” “clubfoot,” “congenital talipes equinovarus,” “case report,” “association,” “cases.” The only relevant article we found in literature was written by Munns et al,^[8]^ who were the only ones to describe a similar case of a mother with OI who gave birth to a child with clubfoot and OI. It is true that OI is one of the commonest genetic disorders of the bone, even if it is a rare disease, but is very uncommon to find it combined with congenital clubfoot.

Munns hypothesized, in his specific case, that the mother's exposure to her periodic therapy with pamidronate may have been the causative factor that generated the clubfoot, even though the administration of this drug had been suspended during her pregnancy. The doses given in this study refer to about 9 mg/kg/year of pamidronate, administered to the mother as drug therapy from the age of 12 to 17, which is when she became pregnant.

Our pharmacological protocol makes use of neridronate, administered intravenously at doses of 2 mg/kg every 3 months, a similar amount to that described by Munns in his study.

In analyzing our case, the mother reported to have suspended the drug treatment, on her own, as of 2 years before her last pregnancy. Therefore, we cannot say with certainty that one of the possible causes of clubfoot in OI can be attributed to the drug treatment with bisphosphonates.

Including the case we just described, the only two cases so far reported in literature of clubfoot combined with OI are both connected to children of mothers with OI. We cannot exclude a multi-factorial etiopathogenesis where the exposure to bisphosphonates also plays a role. In a study conducted by D’Eufemia et al,^[9]^ it is described that, even in the case of an interruption in the administration of neridronate, the drug remains in the circulation of patient's organism for over 2 years. In our case, this could have been the timeline in which our patient was exposed, during pregnancy, to doses of bisphosphonate that were sufficient enough to generate the clubfoot, despite the therapy interruption by part of the mother.

In the case described, the etiology of the clubfoot could be attributed not only to the possible teratogenic effect of neridronate, but also to additional factors. The mother, in fact, reported a history of 3 bouts of polyhydramnios during the pregnancy. During the first bout of polyhydramnios the clubfoot had not yet been diagnosed through the morphological prenatal ultrasound. In our opinion, both the amniocentesis performed on the woman during the 14th and the 20th week of her pregnancy, to drain the amniotic fluid excess, played a role in developing child's clubfoot. Bacino and Hecht^[10]^ recently analyzed and updated the pathogenic theories about clubfoot. They describe the existence of a syndromic and a non-syndromic clubfoot. The syndromic clubfoot is caused by different genetic mutations and is always associated with other genetic disorders, such as atrophy and myotonic dystrophy, distal arthrogryposis, myelomeningocele, or spina bifida. The authors, instead, also describe the non-syndromic clubfoot in their study, which is caused by environmental or prenatal mechanical factors, especially maternal smoking during pregnancy, prenatal fetal exposition to teratogenic drugs, or early amniocentesis (11–14 weeks of pregnancy).

In our opinion, our patient's clubfoot could be classified as non-syndromic, because its combination with OI does not necessarily belong to genetic mutations connected to the basic genetic disorder, but to the fact that the mother underwent two amniocentesis, once during her 14th week of pregnancy and the second one at the beginning of the 20th week. CTE was diagnosed on 23rd week of pregnancy through morphological prenatal ultrasound scan.

In 1980, Hall and Spranger^[11]^ already reported 13 cases of undefined and unclassifiable skeletal dysplasias diagnosed in infants during their examination at birth, defined by the authors as “Congenital Bowing of the Long Bones,” which were characterized most of all by the following different congenital deformities patterns: clubfoot (talipes equinovarus), bowing and deformities of long bones, macrocephalia, microsomia, low weight and/or low height, triangular face, perinatal bone fractures, micrognathia, discoloration/defective teeth. At least three of those patterns were described in each patient reported in the study, and a pregnancy characterized by polyhydramnios, olygohydramnios, or decreased fetal activity was described in almost in all the cases. The authors describe those phenotypes as aspecific bone dysplasias, but are not able to give a certain diagnosis or explain about the exact etiopathogenesis that could have caused the disorders associated to those dysplasias.

In our specific case, as abnormalities associated to the OI type IV, the child presented only light grey sclerae, a mild dentinogenesis imperfecta of the milk teeth, extreme ligamentous laxity, and no hypoacusis. During the physical examination at birth, the patient did not present any visceral defect and there was not yet any evidence of bone deformities. There were no other rare combined congenital disorders to be reported. We searched for disraphisms, spina bifida, congenital hip dislocation, muscular dystrophies, neurofibromatosis, but also for Ehlers–Danlos disease, Marfan syndrome, and any other rare and genetic disease connected to collagen defect.

Now the child is 2 years old, has been bearing weight on his legs since he was 20 months old, and started presenting with the following deformity patterns: left tibia and fibula varus and procurvatus bows, right tibia and fibula procurvatus bow and right femur varus bow. At the moment these deformities are not severe. We frequently evaluate our patients affected by OI, following them during their growth, and we promptly plan the specific surgical correction of deformities when it is necessary.

We first decided to perform a conservative treatment of the clubfoot, by placing lightweight femoro-podalic corrective casts, according to the Ponseti method. Considering that the child was also affected by OI, we planned to manage this treatment carefully given the patient's bone weakness, and to avoid the surgical correction, even though the clubfoot was a really severe type.

To avoid the surgical correction of the most severe clubfoot types, Ponseti et al^[12]^ described a modified conservative treatment, still based on progressive castings to correct the CTE, but for longer periods and with more complex casts to place on children. The results evidenced with this technique were successful.

In this case, we did not expect to be faced with the complications already described above and we could not wait any longer for the surgical correction of the clubfoot, even though the patient was only 3 months old at the moment of the intervention, and that it could be considered reprehensible to perform surgery on such a young infant. However, to avoid any other unexpected problems, because of the patient's fragility caused by OI and the bad compliance of his parents with our cares, we planned to perform the surgery as soon as possible.

A bilateral posteromedial surgical release was performed to correct the clubfoot, using a modified technique consisting in the lengthening of the Achilles tendon, the posterior tibial tendons, the flexor digitorum communis and flexor halluces and the opening of the subtalar, talonavicular and medial cuneiform joints. Edmondson et al^[13]^ also described that, in severely deformed clubfeet, it is correct to perform this surgical technique before 3 months of age, as patients operated before that age have the best results in terms of correction of deformity and functional outcomes in a long-term follow up, compared with those who are more than 3 months old at the time of surgery.

The case we reported is rare among the rare, for that reason, the treatment undertaken was decided not only by carefully assessing the risks associated with the base genetic condition, but also in relation to the unexpected problems we encountered during the course of treatment, which contributed in making the treatment more complex to manage.

In conclusion, in the presence of an orthopedic pathology associated with OI, we recommend always to manage the patient according to the underlying pathology, considering the bone fragility associated with OI at all times. In order to avoid fractures of the OI patient's weak bones, it is important to always place the most lightweight casts, acting with caution and even monitoring any possible autonomous initiative by part of the parents in the management of the case. As was observed by our experience, the final surgical treatment to correct the clubfoot can be successful even if it is done earlier, if necessary, also if the patient is only 3 months old.

Finally, we need to consider a possible association of clubfoot in OI caused by multifactorial factors and not necessarily included in a singular uncommon genetic syndrome. In our case, we hypothesize that the mechanical effect of both the amniocentesis, performed on the mother during pregnancy to drain bouts of polyhydramnios, and the doses of bisphosphonates (which are teratogenic drugs), administered to the mother before conception or during pregnancy, interacting with an already altered genetic substratum, could have played an important role on the clubfoot etiopathogenesis, independently from the genetic mutations or patterns associated with OI type IV. This causality so far has not been established, due to the rarity of the specific case, but it should be analyzed if other cases were to occur in the future, in combination with any other factors that may intervene in the etiopathogenesis of clubfoot in patients with OI.
